# Nanosized Contrast Agents in Ultrasound Molecular Imaging

**DOI:** 10.3389/fbioe.2021.758084

**Published:** 2021-11-29

**Authors:** Fengyi Zeng, Meng Du, Zhiyi Chen

**Affiliations:** ^1^ The First Affiliated Hospital, Medical Imaging Centre, Hengyang Medical School, University of South China, Hengyang, China; ^2^ Institute of Medical Imaging, Hengyang Medical School, University of South China, Hengyang, China; ^3^ Laboratory of Ultrasound Molecular Imaging, The Third Affiliated Hospital of Guangzhou Medical University, Guangzhou, China

**Keywords:** nanosized, contrast agents, ultrasound molecular imaging, nanobubbles, gas vesicles

## Abstract

Applying nanosized ultrasound contrast agents (nUCAs) in molecular imaging has received considerable attention. nUCAs have been instrumental in ultrasound molecular imaging to enhance sensitivity, identification, and quantification. nUCAs can achieve high performance in molecular imaging, which was influenced by synthetic formulations and size. This review presents an overview of nUCAs from different synthetic formulations with a discussion on imaging and detection technology. Then we also review the progress of nUCAs in preclinical application and highlight the recent challenges of nUCAs.

## Introduction

Nanosized ultrasound contrast agents (nUCAs), as a complementary of microbubbles, are developed as contrast enhancers for ultrasound molecular imaging with the ability of penetrating through vasculature for extravascular imaging (Krupka et al., 2010; Wang et al., 2017). High accumulation of nUCAs in an examined area can enhance the signal of target regions with prolonging persistence time compared with microsized UCAs, especially in tumors with the effect of enhanced permeability and retention effect (EPR) (Omata et al., 2020; Perera et al., 2020). Furthermore, the persistence time of the nUCAs lasted obviously longer by binging targeting ligands with receptors in target regions (Jiang et al., 2016; Wang et al., 2018). nUCAs can be modified at their surfaces with specific targeting ligands to improve accumulation in tissues, reduce off-target effect, and improve safety, which is considered as a promising approach in clinical practice.

**FIGURE 1:**
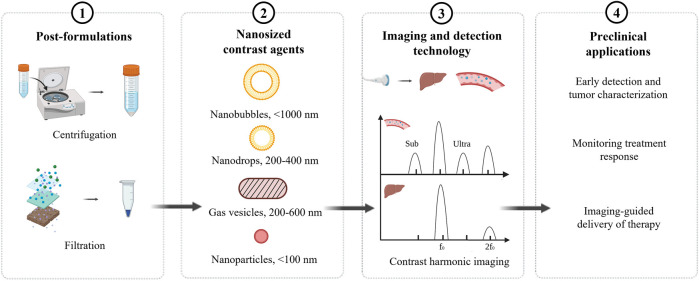
Overview of nanosized contrast agents.

To date, the existing formulations of nUCAs are post formulations of microbubbles, such as centrifuge and filtration. However, post formulations may influence the stability of nUCAs (Tong et al., 2013). Notably, the echogenicity of nUCAs under ultrasound may decrease and show low ultrasound signal because of low backscatter (Gorce et al., 2000; Sheeran et al., 2013). A variety of nUCAs have been developed to improve stability and echogenicity. In this review, the imaging and detection technology is first discussed to further explain the low echogenicity of nUCAs. Then different synthetic formulations of nUCAs will be introduced. While many of these contrast agents are employed for both imaging and therapeutic applications in preclinic, the focus of this review will be more toward their utility and potential as molecular ultrasound imaging agents (Figure 1).

## Imaging and detection technology

Ultrasound imaging has been widely used in clinical fields because of relatively low price, no need of radiation, and noninvasiveness. The principle of early ultrasound imaging is linear imaging, but ultrasonic pulse through tissues and contrast agents would, respectively, produce nonlinear propagation and nonlinear vibration to produce nonlinear acoustic signal (harmonic signal). In the late 1990s, harmonic signals generated by the nonlinear effects of tissues and contrast agents have been used in ultrasound imaging. According to the different sources of harmonic generation, harmonic imaging can be divided into tissue harmonic imaging and contrast harmonic imaging. A variety of experimental studies show that harmonic imaging has better spatial resolution, higher contrast, and clearer image sharpness.

When the pressure of ultrasonic pulse has reached a certain threshold, the frequency of the emitted wave is double than that of ultrasound contrast agents, which can produce a second harmonic signal and ultraharmonic signal to achieve a better imaging. If the frequency fails to achieve threshold, subharmonics and ultraharmonics will still be produced, but the effect is not obvious. Notably, tissue does not produce subharmonics and ultraharmonics. This further improves the contrast between tissue and contrast agents. However, the size of contrast agents has significant influence on the signal; a smaller size, such as NBs, may produce a low signal. In clinical practice, harmonic imaging contains fundamental wave and harmonic wave. The intensity of harmonic wave is much weaker than the fundamental wave, and the signal-to-noise ratio is low. Thus, it requires high sensitivity of equipment to detect and increase the difficulty of equipment development.

Harmonic imaging can be used to detect the signal of contrast agents, but it has a low value to differentiate the signal of nontargeted and targeted contrast agents. Lindner et al. showed that ultrasound signals first contain tissue signals, targeted and freely circulating UCAs, and freely circulating UCAs could be cleared after the cycle time (5–10 min later), and then a high powered ultrasound pulse was applied to destroy the UCAs in the examined area. The difference in signals before and after destruction would be expressed as targeted UCA signals (Lindner et al., 2001; Yu et al., 2019). Another approach to identify targeted contrast agents is evaluating the residence time in an examined area by algorithm. Only ultrasound contrast agents with a dwell time longer than time threshold were regarded as targeted contrast agents. This technology has the advantages of real-time image acquisition without the need to apply high-powered ultrasound (Pysz et al., 2012; Zlitni and Gambhir, 2018). In addition, sensitive particle acoustic quantification technology has been used to quantify receptor expression levels *in vivo* (Wei et al., 1998).

## Chemical synthesis of nanosized ultrasound contrast agents

Nanobubbles (NBs) are nanoparticles (in the nanometer range) that are commonly composed of an encapsulating shell and gas core (Wang et al., 2021). According to different diameters, NBs can be divided into surface and bulk nanobubbles (Craig, 2010; Azevedo et al., 2019). NBs have inferior oscillation behavior relative to microbubbles but are of interest in therapeutic approaches (Ignee et al., 2016). Thus, phase-change contrast agents (PCCAs) have been developed to overcome the size limitation of microbubbles, which can change liquid core into gas core to improve signal-to-noise ratio after activating by ultrasound.

The encapsulating shell mainly influences stability and durability, while the gas determines solubility and acoustic properties (Paefgen et al., 2015). The shell consists mostly of surfactants, polymers, or proteins, while the gas core components are comprised of elevated molecular weights and low solubility filling gases, such as SF_6_ or C_3_F_8_ (Abenojar et al., 2020). To overcome the weak echogenicity of nanobubbles, novel methods are constantly emerging (Wang et al., 2021).

### Encapsulating shell

The shell serves as a barrier to the dissipation of gas between the encapsulated gas and the underlying aqueous medium (Unga and Hashida, 2014). The shell materials are mostly phospholipids or proteins, which are more susceptible to acoustic waves than the hard shells of polymers (Pote et al., 2021). In addition, chitosan is a material of choice for the nanobubble shell because of its low toxicity, low immunogenicity, and excellent biocompatibility (Liu et al., 2021). NBs can be bioconjugated with different forms of drugs or proteins/DNA for selective delivery. Poly(lactic-co-glycolic) acid (PLGA) becomes the preferred choice of pharmaceutical carrier material because of high stability, biodegradability, decreased systemic toxicity, and *in vivo* biocompatibility (Yan et al., 2018). It is a type of polymer synthesized by the polymerization of lactic and glycolic acid in a certain bimolecular weight of the two polymers. In ultrasound molecular imaging, adequate selection of shell materials is critical for various applications with different rigidities, charges, thicknesses, and functional groups. To improve the echogenicity of nanobubbles for extravascular imaging, Exner et al. have reported on the formulation of echogenic perfluoropropane gas nanobubbles stabilized by a lipid–pluronic surfactant shell (Hernandez et al., 2017). Furthermore, they describe a novel nanobubble of perfluoropropane gas stabilized by a surfactant and lipid membrane and a crosslinked network of N,N-diethylacrylamide (Perera et al., 2017; de Leon et al., 2019). These results demonstrate the capabilities and advantages of a new, more stable, nanometer-scale ultrasound contrast agent that can be utilized in future work for diagnostic scans and molecular imaging.

### Gas core

The type of gas core determines its residence time in systemic circulation. In ultrasound molecular imaging, hydrophobic gases are typically employed because they are immiscible in the aqueous environment, which prevents them from leaking out fast and leads to a longer bubble half-life. Among hydrophobic gases, those with higher molecular weight, higher gas density, and lower diffusivity coefficient are expected to give more stable bubbles (Kanbar et al., 2017; Omata et al., 2019). Perfluorocarbons (PFCs) are biocompatible, biologically inert, and highly stable chemicals that are not metabolized in the body after injection (Schutt et al., 2003; Cheng, 2004). In addition, increasing the chain length of PFCs by CF_2_ leads to an order of magnitude decrease in solubility in water (Chomas et al., 2001; Klibanov, 2002). PFCs also reduce interfacial tension, which can further improve bubble performance (Nguyen et al., 2011; Hernandez et al., 2018). Thus, a large amount of research has focused on using heavier perfluorocarbons, such as C_4_F_10_, C_5_F_12_, and C_6_F_14_ (Sheeran and Dayton, 2012).

## Biogenic synthesis of nanosized ultrasound contrast agents

The stability of nanobubbles has raised questions because of their surface tension forces. In addition, the safety of nanobubbles is also controversial because of their synthetic materials and chemical formulation. Shapiro et al. obtained biogenic gas nanobubbles derived from two different microorganism species for molecular imaging, which are named as gas vesicles (GVs) (Shapiro et al., 2014). Gas vesicles were encoded in many bacteria and phyla of archaea, which were illustrated in previous reviews (Pfeifer, 2012; Garrute and Machado, 2020). For this reason, we chose to review the research that used GVs as ultrasound molecular reporters.

Gas vesicles have a protein shell with a hollow gas-filled core, with a dimension of ~200 nm and a thickness of ~2 nm. GVs are encoded by 8–14 genes, including the primary structural proteins GvpA and GvpC, and several secondary proteins that function as essential minor constituents or chaperones (Pfeifer, 2012). GvpA is a 7.4-kDa amphiphilic protein that serves as the main structural backbone of the GV shell by forming 4.6-nm-wide ribs, while GvpC is a protein that provides structural reinforcement (Walsby and Hayes, 1989; Buchholz et al., 1993). Shapiro et al. found that the removal, addition, or modification of GvpC would alter the acoustic properties of GVs (Lakshmanan et al., 2016). With the development of genetic engineering, plasmids with gvp genes were transferred into engineered bacteria to produce GVs, which can signify cellular location and function (Bourdeau et al., 2018; Huang et al., 2019) (Table 1).

**TABLE 1:** The production of gas vesicles (GVs) from engineered bacteria in ultrasound imaging (Bourdeau et al., 2018; Huang et al., 2019).

Engineered bacteria	Plasmids	Promoter	Induced
*Escherichia coli BL21(A1)*	pET28a_T7-ARG1	T7	0.5% l-arabinose and 0.4 mM IPTG
*E. coli Nissle 1917*	pET28a_T5-ARG1	T5	3 μM IPTG
*Salmonella typhimurium* ELH1301	pTD103	PLUX	3 nM AHL
*Streptomyces*	pSET152-gvp3234	ermE	NA
*Serratia* sp. *ATCC 39006*	pET28a	T7	0.1% l-arabinose

## Targeted strategies of nanosized ultrasound contrast agents

Ultrasound molecular imaging relies on delivering UCAs to a specific site. There are two different classification methods of strategies involving the coupling of UCAs for targeting: 1) passive targeting or 2) active targeting (Table 2).

**TABLE 2:** Different modifications of nanobubbles (Jiang et al., 2015; Yang et al., 2016; Cai et al., 2018; Peng et al., 2019; Wu et al., 2019; Fang et al., 2020; Jiang et al., 2020; Li et al., 2020; Zhang et al., 2020).

Pre formulation	Post formulation	Diameter	Targets	Binding ligands	Modification	Application
Thin-film hydration	Centrifugation	472.9 ± 60.3 nm	AMD070	CXCR4	EDC and NHS	Imaging Jiang et al. (2015)
Thin-film hydration and sonication	Centrifugation	613.0 ± 25.4 nm	HER2	HER2-antibody	EDC and NHS	Imaging Yang et al. (2016)
Thin-film hydration	Centrifugation	428.0 ± 12.5 nm	CSF-1R	CSF-1R-antibody	Biotin streptavidin	Imaging Cai et al. (2018)
Membrane hydration and mechanical vibration	Centrifugation	459.3 ± 37.0 nm	Nucleoli	AS1411	EDC and NHS	Imaging Peng et al. (2019)
Thin-film hydration	Centrifugation	549.33 ± 28.53 nm	Anti-Müllerian	AMH antibody	Biotin avidin	Imaging Wu et al. (2019)
Thin-film hydration	Centrifugation	442.5 ± 48.6 nm	Tumor cells	IR-780	Liposoluble	Delivery Fang et al. (2020)
Thin-film hydration	Centrifugation	625.4 ± 63.8 nm	-	-	Streptavidin	Delivery Jiang et al. (2020)
Thin-film hydration and mechanical sonication	Filtration	427.7 ± 84.8 nm	Tumor cells	anti-GPC3 antibody	Biotin avidin	Imaging and Delivery Li et al. (2020)
Double emulsion (water/oil/water)	Filtration	525 ± 173 nm to	CAIX	CAIX antibody	EDC and NHS	Monitoring Zhang et al. (2020)
evaporation		694 ± 282 nm				

## Passive targeting

Passive targeting is related to the so-called enhanced permeability and retention (EPR) effect, which is a feature of many tumors and diverse inflammation sites. The pathological changes in blood vessels and normal blood vessels on the structure and morphology is different, the gap between the endothelial cells can be widened to 800 nm, and lymph is blocked at the same time. These two major reasons prompt nUCAs to seep in the enhancement of the lesion site and retention.

## Active targeting

Active targeting includes noncovalent conjugation and covalent conjugation. Noncovalent conjugation is similar to the modification and conjugation of ligands. Incorporation of phosphatidylserine in the shell of NBs results in activation and surface attachment of complement fragments because of a highly negative charged shell (Lindner et al., 2000a). In addition, covalent conjugation needs EDC and NHS to activate functional groups and active-carboxyl groups (Jiang et al., 2015; Peng et al., 2019; Fang et al., 2020; Li et al., 2020), while coupling can also be accomplished *via* thiol-maleimide and coupled to a thiol-activated ligand. Albumin-shelled NBs also can bind to activated leukocytes *via* β2-integrins on leukocytes (Lindner et al., 2000b; Lindner et al., 2000c) and complement fragments (Anderson et al., 2007). In a more specific approach to targeting, different ligands such as antibody, peptides, and glycoproteins are conjugated to the shell surface. Additionally, the properties of targeted agent, hemodynamic, and target molecule are the major determinants for targeted UCA retention in areas of diseases (Brown and Lindner, 2019). To reduce the recognition by the immune system and increase half-life in circulation, the PEG spacer is used to conjugate with the targeting agent (Borden et al., 2013).

## Preclinical application of nanosized ultrasound contrast agents

Ultrasound molecular imaging could potentially be used in early detection, monitoring treatment effects, and delivery of drugs. To date, there are various preclinical applications to explore the effect of targeted nUCAs (Wei et al., 1998; Yin et al., 2012).

### Early detection and tumor characteristic

New effective targets overexpressed on tumor tissue and neovasculature are regarded as a breakthrough for early accurate diagnosis and characteristic. Guo et al. constructed lipid nanobubbles combined with AS1411 to highly target nucleolin in triple-negative breast cancer, which can realize molecular imaging of tumor tissues and neovasculature to provide an early detection method (Li et al., 2020). In addition, CA-125-targeted echogenic lipid and surfactant-stabilized nanobubbles were developed to enhance tumor accumulation, which may contribute to improved diagnosis of epithelial ovarian cancer (Gao et al., 2017). Additionally, targeted NBs linked to CSF-1R have been shown to successfully target the margin of hepatocarcinoma, thereby improving the efficiency of radiofrequency ablation (Wu et al., 2019). In addition, targeted NBs have been shown to target a variety of skin-derived tumors *in vitro* using PLGA-linked NBs to target residual tumors (Xu et al., 2010; Zhang et al., 2014), as an adjunct to synergistic radiofrequency ablation under high-intensity focused ultrasound (HIFU) (Watanabe et al., 2010; Perera et al., 2014). Using the energy of contrast agent rupture can significantly enhance the apoptosis of tumor cells. Some scholars successfully detected the early inflammatory response stage of atherosclerotic plaques by preparing a nanoscale UCA with magnetic targeting of VCAM-1 (Deshpande et al., 2016).

### Monitoring treatment response

The expression level of the target molecules, which reflects the diseased or abnormal status, can be evaluated *in vivo* by quantitative analysis of the ultrasound contrast signal intensity. In order to monitor the survival of ovarian cells in the early transplantation, MU et al. developed AMH-targeted nanobubbles by integrating an AMH antibody onto the surface of NBs. Evidence showed that the ultrasound signal was relative with the expression of AMH in transplant time (Lindner et al., 2000b). In addition, targeted nanobubbles have been used to monitor tamoxifen resistance through the expression of carbonic anhydrase IX in breast cancer (Borden et al., 2013).

### Imaging-guided delivery of therapy

Recently, NBs have been researched with regard to the efficient delivery of exogenous genes and drugs noninvasively (Maxim et al., 2019). A recent trend is that ultrasound-targeted NB destruction (UTND) plays a crucial role in improving the efficient delivery by sonoporation of NBs (Roberta et al., 2013; Li et al., 2021). Under ultrasound, cell membrane permeabilization and enhancing drug uptake can be caused by NBs. Notably, nontargeted NBs are readily swallowed by the reticuloendothelial system, thereby reducing the aggregation of target areas. Therefore, it is necessary to develop targeted and drug-loaded NBs to improve therapeutic effect and reduce off-target effect (Pote et al., 2021). Drug-loaded phase-transformation lipid nanoparticles are a promising drug carrier that can provide both physical and chemical therapy in combination with ultrasound for molecular imaging and therapy (Li et al., 2018; Zhu et al., 2018). Zhu et al. have prepared phase-transformation lipid nanoparticles with paclitaxel loaded and anti-LHRHR targeted. This drug carrier can actively target and specifically kill ovarian-3 cells. At the same time, it can occur in liquid–gas phase-transformation under low-intensity focused ultrasound to enhance the ultrasound imaging (Gao et al., 2018). Additionally, nanobubbles combined with ultrasound-targeted destruction (UTD) have become potential carriers for gene delivery (Cai et al., 2018). The NB-siRNA nanoparticle was used to target NB-siRNA to improve siRNA transfection under ultrasound irradiation, which is effectively enhancing the effect of siRNA transfection and *in vitro* silencing of targeted genes (Maxim et al., 2019).

## Challenges of nanosized ultrasound contrast agents

Microsized ultrasound contrast agents have been commercialized and used in diagnosis and adjuvant treatment with less and mild adverse reactions. In contrast, the research of nUCAs have been widely explored (Liufu et al., 2020), while rarely used in clinical application. The main problems of the research are as follows. The decrease in signal intensity, the decrease in inner diameter of contrast agents, and the decrease in backscattering ability are urgent problems to be solved in the research of nUCAs. It is difficult to accurately control the size of nUCAs that not only cross the vasculature but also have a strong scattering performance. In addition, the shell and core of the nUCAs will affect the stability and residence time. Thus, how to reduce the size of nUCAs and increase the signal becomes the focus of future research. The low concentration of target tissue aggregation has been confirmed by many experiments, and how to choose the best ligand and receptor needs to be further explored. Besides the influence of nUCAs materials on normal tissue, the accuracy of lesion location and the optimization of ultrasonic instrument parameters need to be further explored. With the continuous cross fusion of biomedical and clinical medical technology, the existing problems of nano contrast agents will be solved continuously, and the safety will be improved. It is believed that ultrasound contrast agents will play an irreplaceable role in the early diagnosis and accurate treatment of clinical diseases in the future.

## Future perspective

The feasibility of ultrasound molecular imaging studies has been demonstrated in numerous preclinical studies and used in different disease models. Therefore, ultrasound molecular imaging has been used in understanding the progression of disease mechanisms, which has also been used in preclinical testing of the efficacy of new drugs. This field is expected to expand with better standardization of target contrast agents and ultrasound imaging protocols. As more and more targeted nanosized ultrasound contrast agents are developed, the ability to characterize specific disease phenotypes will become critical. However, the widespread use of nUCAs in the clinic will depend on additional, extensive, and larger clinical trials that demonstrate safety. In addition, the integration of clinical information obtained from molecular imaging will need to be integrated into diagnostic and therapeutic pathways to improve diagnostic accuracy.

## Executive summary

Nanosized ultrasound contrast agents have been successfully used in early diagnosis and response to therapy.

Nanosized ultrasound contrast agents have several challenges, such as the decrease in signal intensity, complex preparation process, the low concentration in the targeted area, and so on.
